# Highly Efficient Photochemical Vapor Generation for Sensitive Determination of Iridium by Inductively Coupled Plasma Mass Spectrometry

**DOI:** 10.1021/acs.analchem.2c04660

**Published:** 2023-02-10

**Authors:** Stanislav Musil, Eva Jeníková, Jaromír Vyhnanovský, Ralph E. Sturgeon

**Affiliations:** † Institute of Analytical Chemistry of the Czech Academy of Sciences, Veveří 97, 602 00 Brno, Czech Republic; ‡ Faculty of Science, Department of Analytical Chemistry, Charles University, Hlavova 8, 128 43 Prague, Czech Republic; § National Research Council of Canada, 1200 Montreal Road, Ottawa, Ontario K1A 0R6, Canada

## Abstract

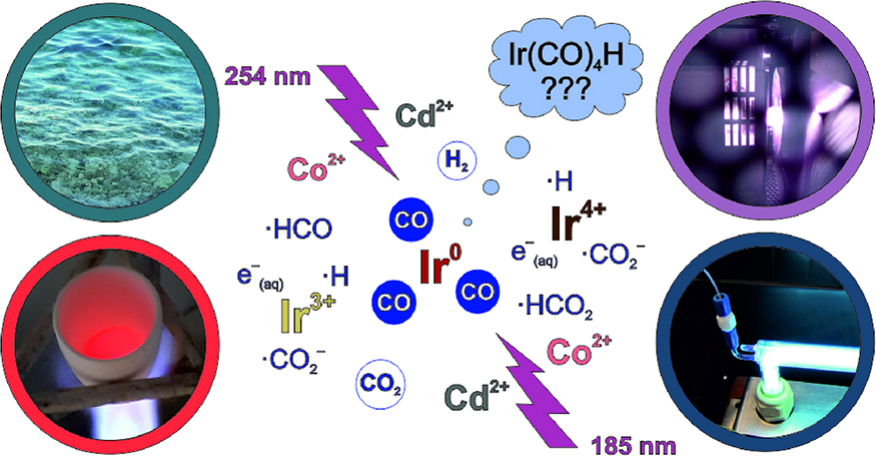

Herein, we describe the highly efficient photochemical vapor generation (PVG) of a volatile species of Ir (presumably iridium tetracarbonyl hydride) for subsequent detection by inductively coupled plasma mass spectrometry (ICPMS). A thin-film flow-through photoreactor, operated in flow injection mode, provided high efficiency following optimization of identified key PVG parameters, notably, irradiation time, pH of the reaction medium, and the presence of metal sensitizers. For routine use and analytical application, PVG conditions comprising 4 M formic acid as the reaction medium, the presence of 10 mg L^–1^ Co^2+^ and 25 mg L^–1^ Cd^2+^ as added sensitizers, and an irradiation time of 29 s were chosen. An almost 90% overall PVG efficiency for both Ir^3+^ and Ir^4+^ oxidation states was accompanied by excellent repeatability of 1.0% (*n* = 15) of the peak area response from a 50 ng L^–1^ Ir standard. Limits of detection ranged from 3 to 6 pg L^–1^ (1.5–3 fg absolute), dependent on use of the ICPMS reaction/collision cell. Interferences from several transition metals and metalloids as well as inorganic acids and their anions were investigated, and outstanding tolerance toward chloride was found. Accuracy of the developed methodology was verified by analysis of NIST SRM 2556 (Used Auto Catalyst) following peroxide fusion for sample preparation. Practical application was further demonstrated by the direct analysis of spring water, river water, lake water, and two seawater samples with around 100% spike recovery and no sample preparation except the addition of formic acid and the sensitizers.

## Introduction

Iridium belongs to the platinum group metals (PGMs) and is one of the rarest elements on Earth. For example, the concentration of dissolved Ir in seawater was reported to be in the range 0.1–10 pg L^–1^.^1,2^ It is relatively abundant in meteorites, and some natural enrichment at the earth’s surface occurs due to volcanic activity.^3^ Distinctive properties include its resistance to chemical attack, excellent high-temperature characteristics, and electrical properties. These are increasingly exploited for industrial applications in the electronic, chemical, and automotive industries in the form of alloys with Pt, Pd, and Rh for automotive catalytic converters, in crucibles, thermocouples, spaceship engines, gas turbines, and spark plugs.^4,5^ Its increasing use has also raised the issue of its impact on biogeochemical and environmental cycles^6^ as well as human health concerns, driving the demand for analytical methodologies providing baseline data to establish natural levels from which assessment of changes can be reliably made.^5^

Determination of extremely low concentrations of Ir in the environment poses a considerable analytical challenge. Overviews of the main analytical methodologies and sample preparation scenarios, including various preconcentration techniques, to assess such low Ir levels have recently become available.^4,7^ Among the variety of techniques, inductively coupled plasma mass spectrometry (ICPMS) has gained a particular popularity due to the speed of analysis and extreme sensitivity of determination of many elements. For some elements, a substantial improvement in detection capability may be provided when coupled to vapor generation (VG) for analyte introduction due to their conversion to a volatile compound (species), separation from the liquid matrix, and efficient gas phase transport to the detector. VG can offer substantially higher analyte introduction efficiency (100% in an ideal case) than is typical for conventional solution pneumatic nebulization (PN, <10%). Other benefits arise due to the removal of the sample matrix in that the analyte is often selectively separated from the liquid phase, eliminating spectral and non-spectral interferences during detection and further contributing to enhanced detection power.

Compared to the mature approach of VG based on chemical reduction of analyte by tetrahydridoborate, with photochemical vapor generation (PVG), volatile analyte species are synthesized during UV irradiation of an aqueous reaction medium typically containing low-molar mass carboxylic acids such as formic and acetic acid.^8,9^ The resulting highly reducing radical species (H^•^, CO_2_^•–^), and e_(*aq*)_^–^), along with photogenerated R^•^ and CO, subsequently interact with the analyte to form volatile hydrides, carbonyls, or alkylated compounds depending on the analyte and reaction medium used. PVG is still rapidly developing in scope, having added more than 10 new elements in the past 5 years.^10−19^ This is also due to the recent introduction of metal sensitizers that significantly increase the PVG efficiency or are even indispensable to initiate PVG of some analytes. Metal sensitizers exhibiting such effects on PVG include: Cd^2+^ ions for As^3+^ and As^5+^,^20^ Se^6+^,^21^ and W^6+^;^15^ Co^2+^ ions for Bi^3+^,^22,23^ Cd^2+^,^24^ Ge^4+^,^10^ Pb^2+^,^25^ Sb^3+^,^26^ Te^4+^,^27^ and Tl^+^;^28^ Cu^2+^ ions for Br^–^,^29^ Cl^–^,^19,29^ F^–^,^14,29^ Ir^3+^,^17^ and Rh^3+^;^17^ Fe^2+^ and/or Fe^3+^ ions for As^3+^,^30^ Bi^3+^,^31^ Te^4+^,^32^ Mo^6+^,^16^ Cd^2+^,^33^ and Os^4+^;^18^ Ni^2+^ ions for Pb^2+^;^25^ and V^4+^ and V^5+^ for Te^4+^ and Te^6+^.^34^ A greater effect (additive or synergistic) was reported for several analytes when a combination of two metal sensitizers was employed: namely, Co^2+^ and Cu^2+^ ions for Mo^6+^;^35^ Cd^2+^ and Co^2+^ for Os^4+^,^12^ Re^7+^,^11^ and Ru^3+^;^12,13^ and Mn^2+^ and Fe^2+^ ions for Te^4+^.^36^ The mechanism of the action of the metal sensitizers on PVG remains to be fully elucidated, but the latest studies dealing with application of electron paramagnetic resonance spin trapping techniques support the initial thesis^8^ that transition metal ions enhance the yield of highly reducing CO_2_^•–^ during UV irradiation of HCOOH.^11,27,34,35,37^

The feasibility of PVG of Ir was demonstrated by de Oliveira and Borges^17^ within a multi-element study (also devoted to Au, Pd, Pt, and Rh) based on the use of a comparatively inefficient photoreactor (no vacuum UV capability) consisting of two 40 W low-pressure Hg lamps to irradiate a quartz tube through which the sample was passed. With the main focus on seawater analysis, they identified a significant positive effect of Cu^2+^ ions enhancing PVG yield and attained a 20 ng L^–1^ limit of detection (LOD) by coupling PVG sample introduction with ICPMS. In a recent 17-element mechanistic study by Yu et al.,^37^ the authors simply adopted the same sample generation medium for PVG of Ir but used an advanced high-efficiency flow-through photoreactor with and without introduction of air segments before and after a zone of liquid sample. The authors estimated the PVG efficiency to be in the range of 60–70% by comparison of the Ir signal intensities with those from the PN-ICPMS of solutions before and after their UV irradiation. No LOD was presented.

The current work was undertaken in an effort to identify PVG conditions that would offer highly efficient and repeatable PVG yields useful for ICPMS detection of Ir and to develop an extremely sensitive analytical methodology for its determination at pg L^–1^ levels (ppq).

## Experimental Section

### Instrumentation

The same PVG system described in our recent study of PVG of Ru,^13^ based on a flow injection (FI) mode of operation and coupled to ICPMS, was utilized (see the Supporting Information for a scheme and details on the entire arrangement). Briefly, it consisted of a chemifold fitted with an injection valve (0.5 mL sample loop), a plastic gas–liquid separator (GLS, 15 mL), and a high-efficiency flow-through photoreactor (19 W, Jitian Instruments Co., China). A 200 mL min^–1^ flow of Ar carrier was used to provide the efficient release of volatile Ir species and their transport from the GLS to the ICPMS (see details in Supporting Information).

Detection was achieved using an Agilent 8900 triple-quadrupole ICPMS operating in time-resolved analysis and single-quadrupole modes using either no gas or He gas (4.1 mL min^–1^) in the reaction/collision cell. Optimal plasma settings for PVG measurements and selected isotopes are summarized in Table S1 of the Supporting Information. If not explicitly stated, all results are based on ^193^Ir peak area response. Note that no statistically significant difference in optimized parameters, apart from expected sensitivity, was evident when monitoring ^191^Ir.

### Reagents and Materials

Deionized water (DIW, <0.2 μS cm^–1^, Ultrapur, Watrex) was used for the preparation of all solutions unless otherwise stated. Formic acid (98%, p.a., Lach-Ner, Czech Republic) was used for the formulation of reaction media of various molarities (M, i.e., mol L^–1^). A commercial stock analytical standard solution of 1024 mg L^–1^ Ir^3+^ (as IrCl_3_) in 10% (m/v) HCl was obtained from Sigma-Aldrich. Standard solutions containing 870 mg L^–1^ Ir^3+^ and 815 mg L^–1^ Ir^4+^ were prepared by dissolving solid ammonium hexachloroiridate(III) ((NH_4_)_3_[IrCl_6_], Fluka) and ammonium hexachloroiridate(IV) ((NH_4_)_2_[IrCl_6_], Fluka) in 10% (m/v) HCl. The following compounds were used as potential metal sensitizers for PVG: cadmium(II) acetate dihydrate (p.a., Lach-Ner), cobalt(II) acetate tetrahydrate (99.999%, Alfa Aesar), copper(II) acetate monohydrate (Merck), iron(II) acetate (≥99.99%, Sigma-Aldrich), manganese(II) acetate tetrahydrate (p.a., Sigma-Aldrich), and nickel(II) acetate tetrahydrate (p.a., Sigma-Aldrich). Stock solutions of metal sensitizers were prepared by dissolution of these metal acetates in 0.2% (m/v) CH_3_COOH and contained 2.5–10 g L^–1^ of individual metals. For interference studies, stock solutions of 1000 mg L^–1^ Au^3+^, Fe^3+^, Mn^2+^, and Pt^4+^ in 1 M HCl and 1000 mg L^–1^ Zn^2+^ in 1 M CH_3_COOH were sourced from BDH (UK), 1000 mg L^–1^ Mo^6+^ in 0.5% (m/v) NH_4_OH from Absolute Standards, Inc. (USA), 1000 mg L^–1^ Rh^3+^ in 5% (m/v) HCl from Fluka, 1000 mg L^–1^ Pd^2+^ in 5% (m/v) HCl from Sigma-Aldrich, and 1000 mg L^–1^ Cu^2+^ in 2% (m/v) HCl from Analytika (Czech Republic). A 1000 mg L^–1^ solution of As^3+^ in DIW was prepared from arsenic(III)oxide (Lachema, Czech Republic), and 5000 mg L^–1^ Se^4+^ in DIW was prepared from sodium selenite(IV) pentahydrate (Lachema). Other chemicals were sourced as follows: ammonium hydroxide (25%, p.a.), nitric acid (65%, semiconductor grade), and sodium formate from Sigma-Aldrich; acetic acid (99.8%, p.a.), sulfuric acid (96%, chem. pure), and sodium chloride (p.a.) from Lach-Ner; sodium hydroxide (p.a.) from Penta (Czech Republic); hydrochloric acid (35%, Analpure) and ultrapure water (Analpure Ultra) from Analytika; sodium peroxide (≥95%, p.a.) from Carl Roth; and sodium nitrate (Suprapur) from Merck.

### Procedure and Conventions

Measurements were conducted in the FI mode using a constant flow of the carrier (reaction) medium propelled by a peristaltic pump. A standard/sample prepared in the reaction medium and spiked with selected sensitizers, if any, was manually injected into the carrier stream at the beginning of a PVG cycle of recording signal intensities. Integration of the signal was stopped after the transient signal returned to the baseline.

Peak area (counts) of the flow injection transients, normalized to the averaged signal from a ^185^Re internal standard (IS) simultaneously admitted by PN over the same measurement time window, was employed as a measure of analyte response. Each result is presented as the average of at least three peak area replicates with an uncertainty given as ± 1 standard deviation (SD) or combined uncertainty where results are relative. Overall PVG efficiency is defined as the fraction of the analyte that is converted to volatile species, released to the gas phase, and transported to the plasma. Nebulization efficiency represents the fraction of the analyte introduced into the spray chamber and transported to the plasma. Overall PVG efficiency was determined according to our previously published procedure^13,15,22,33^ and is the product of a sensitivity enhancement factor and absolute nebulization efficiency. The enhancement factor is defined as the ratio of the peak area sensitivity obtained with FI-PVG sample introduction to that arising from FI-PN during their concurrent operation, i.e., both measured under exactly the same plasma conditions. The nebulization efficiency was determined under optimal settings of the ICPMS (Table S1) using a modified waste collection method (sometimes called a dynamic mass flow approach).^38^ To provide an immediate indication of a state of optimization of PVG of Ir (or other elements), the overall PVG efficiency was estimated for each measurement by comparison of peak area sensitivity obtained with FI-PVG and FI-PN. These values are given in the text without a corresponding uncertainty.

For comparison, overall PVG efficiency was also quantified using an indirect approach based on determination of the amount of Ir in the waste solution after PVG using conventional PN-ICPMS (details on this procedure are available in ref (39)).

### Sample Preparation

Five natural water samples comprising various matrices were analyzed. A spring water sample was collected at a chapel of the Bohosudov Church (Krupka u Teplic, Czech Republic); a river water sample was collected from the Vltava River in Prague, and lake water was sampled from the Lipno Reservoir, the largest water feature in the Czech Republic. Additionally, two seawater samples (I and II) were collected at two locations on the Istrian Peninsula in Croatia (Camping Mon Perin and Sveta Marina). Samples were filtered through a 0.45 μm PTFE filter and prepared for parallel analysis by FI-PVG-ICPMS (prepared in 4 M HCOOH containing 10 mg L^–1^ Co^2+^ and 25 mg L^–1^ Cd^2+^) and by conventional PN-ICPMS/MS (prepared in 2% (m/v) HNO_3_).

A pulverized NIST Standard Reference Material 2556 – Used Auto Catalyst (Pellets) was decomposed by means of sodium peroxide fusion. Approximately 0.4 g of SRM 2556 was mixed with 2 g of Na_2_O_2_ in a sintered alumina crucible (27 mL). The heating program was as follows: 15 min at 220 °C followed by 30 min at 620 °C. After cooling, the vitreous sample mass was carefully dissolved in 20 mL of 5 M HCl and 30 mL of water by alternately adding water and 5 M HCl in 5 mL increments. This digest was further diluted and prepared for parallel analysis by FI-PVG-ICPMS (in 4 M HCOOH containing sensitizers) and by conventional PN-ICPMS/MS (in 2% (m/v) HNO_3_).

## Results and Discussion

### PVG without Additives

**Figure 1:**
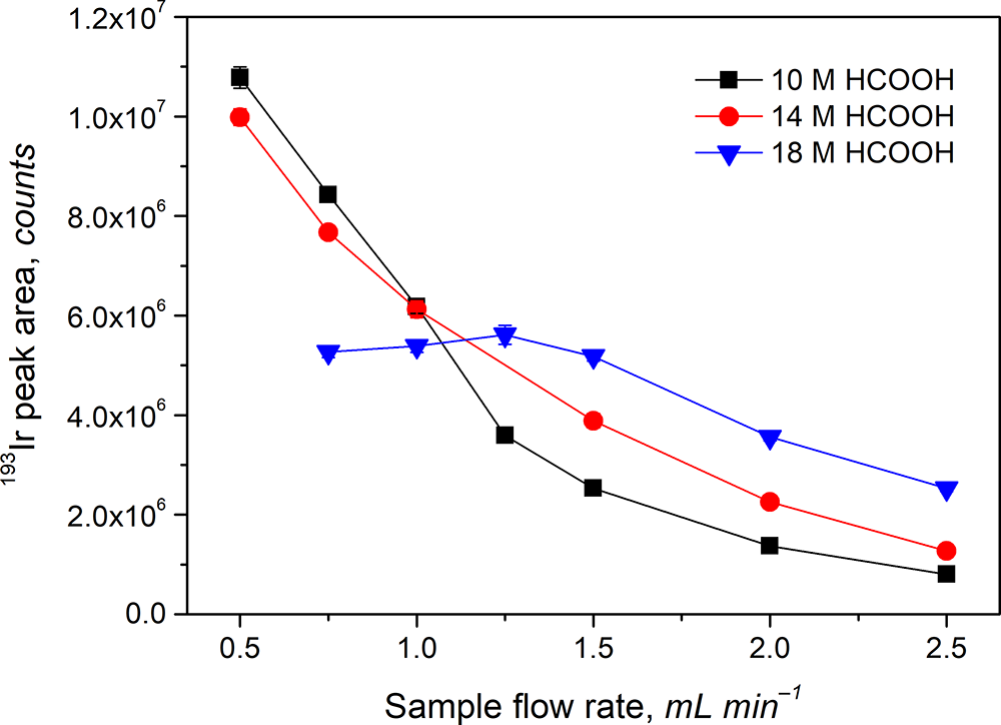
Effect of sample flow rate at various concentrations of HCOOH in the reaction medium on peak area response from 200 ng L^–1^ Ir^3+^. Uncertainties expressed as SD (*n* ≥ 3) are sufficiently small that they cannot be discerned from the data points in some cases.

The PVG reaction was first examined using only an HCOOH medium in the absence of metal ion sensitizers or other additives in order to establish a “baseline” performance. The most important parameters influencing PVG were investigated, including HCOOH concentration and sample irradiation time (IT), which is inversely proportional to the flow rate of the sample through the photoreactor. Using a flow rate of 1.5 mL min^–1^ through the photoreactor (IT = 29 s), the effect of HCOOH concentration was examined in the range of 2–20 M HCOOH. Sensitivity gradually increased with higher HCOOH concentrations and reached a maximum at 18 M HCOOH (see Figure S2 in the Supporting Information). These signals were confirmed to arise from PVG processes because absolutely no signals were detected at 4, 10, and 18 M HCOOH when the UV lamp was not powered. From a comparison of peak area response using a 1 μg L^–1^ solution Ir^3+^ in 2% (m/v) HNO_3_ introduced via the same 500 μL sample loop and FI-PN mode, overall PVG efficiency at 18 M HCOOH was estimated to be ≈9%. The effect of IT was subsequently investigated at three concentrations of HCOOH (10, 14, and 18 M HCOOH) by altering the sample flow rate in the range of 0.5–2.5 mL min^–1^ (Figure 1).

It is evident that for 10 and 14 M HCOOH the sensitivity gradually increases down to a flow rate of 0.5 mL min^–1^ (IT = 87 s) but no maxima are identified in the tested range. The decline at higher flow rates was proven to be due to insufficient irradiation of the sample and not to a lower efficiency of the gas–liquid separation process in the GLS (see experimental results confirming this conclusion in the Supporting Information). The overall PVG efficiency for 10 M HCOOH at 0.5 mL min^–1^, representing the conditions with the highest sensitivity noted in Figure 1, was estimated to be ≈18%. In contrast to 10 and 14 M HCOOH media, the dependence for 18 M HCOOH is different, reaching a maximum at 1.25 mL min^–1^, wherein a slight decline of sensitivities follows at both lower and higher sample flow rates. This can be explained by an increased production of bubbles (comprising CO, CO_2_, and H_2_) from UV homolysis of HCOOH in the photoreactor, clearly observed at flow rates ≤1.25 mL min^–1^. Bubble expansion tends to expel the reaction medium from the photoreactor, and IT likely effectively remains the same in the 0.75–1.25 mL min^–1^ range. No significant bubble formation is observed at any sample flow rate using 10 M HCOOH while their formation was initiated at ≤0.75 mL min^–1^ using 14 M HCOOH. Signals generated from 6, 10, and 14 M CH_3_COOH media at 1.5 mL min^–1^ were 200–250-fold lower than those obtained with 6, 10, and 14 M HCOOH. The addition of 1 or 2 M CH_3_COOH to 9 and 8 M HCOOH also did not provide any enhancement effect on PVG of Ir in comparison to 10 M HCOOH alone. Hence, CH_3_COOH is not a suitable medium for PVG of Ir, consistent with observations on PVG of other transition metals with the exception of Os^18^ and Re.^11^ For the latter, CH_3_COOH was fruitful only when used in combination with HCOOH.

These pilot experiments demonstrated that PVG of Ir necessitates rather high concentrations of HCOOH and long ITs when conducted without additives. Generally, high concentrations of HCOOH are not suitable due to the accompanying dilution of real samples by added HCOOH prior to analysis.^39^ Potential improvement in overall PVG efficiency could possibly be realized at 10 and 14 M HCOOH by further increase in the IT. This, however, is not well tolerated in this flow system because the flow injection peaks become too broad even at flow rates of ≤1 mL min^–1^, requiring long integration times and limitations on sample throughput. In order to substantially enhance the overall PVG efficiency while keeping rather short IT and relatively low HCOOH concentration in the reaction medium, the effect of metal sensitizers or other additives influencing pH was investigated.

### Effect of pH

Similar to PVG of Fe, Co, and Ni,^39−44^ an increase in pH of the reaction medium exerted a significant effect on the PVG efficiency (Figure S3). The effect of pH was examined by varying the volume of added liquid ammonia (NH_3_·H_2_O) to partially neutralize solutions of 10 M HCOOH. The resulting reaction media containing 0–5 M of formed HCOONH_4_ in 5–10 M of “unreacted” HCOOH were used as the carrier and for preparation of the Ir^3+^ standard that was injected into a continuous stream of this carrier medium. A maximum of peak area was reached at pH = 3.4 and 3.7, corresponding to media containing 4 M HCOONH_4_ in 6 M HCOOH and 5 M HCOONH_4_ in 5 M HCOOH, respectively. This provided an approximately 4.5-fold signal increase relative to only 10 M HCOOH (pH = 1.38), corresponding to an overall PVG efficiency of ≈19%. The effect of IT was then examined at pH = 3.4, i.e., using a mixture of 4 M HCOONH_4_ and 6 M HCOOH (Figure S4). Analogous to the reaction media containing only 10 M or 14 M HCOOH (cf. Figure 1), no maximum sensitivity was reached in the range of 0.5–2.5 mL min^–1^ and the sensitivity exponentially increased with lower flow rates and corresponding longer ITs. Nevertheless, the estimated overall efficiency of ≈71% at 0.5 mL min^–1^ suggests the potential for PVG becoming highly efficient.

The same enhancement effect on peak area sensitivity was observed when liquid NH_3_·H_2_O was replaced with solid NaOH to partially neutralize 10 M HCOOH. Interestingly, an overall PVG efficiency of ≈8% can be reached when PVG is conducted from 10 M HCOONa (pH = 9.4) prepared by dissolution of solid HCOONa in DIW, which represents around 2-fold increase in comparison to PVG from 10 M HCOOH (pH = 1.38). It should be noted, however, that the preparation of such reaction media from highly concentrated HCOOH and high volumes of ammonia or large masses of NaOH is laborious as the solutions need to be cooled during preparation, and it also introduces a high risk of contamination. Due to such limitations, pH adjustment of the reaction medium was not undertaken with any subsequent experiments.

### PVG in the Presence of Metal Ion Sensitizers

**Figure 2:**
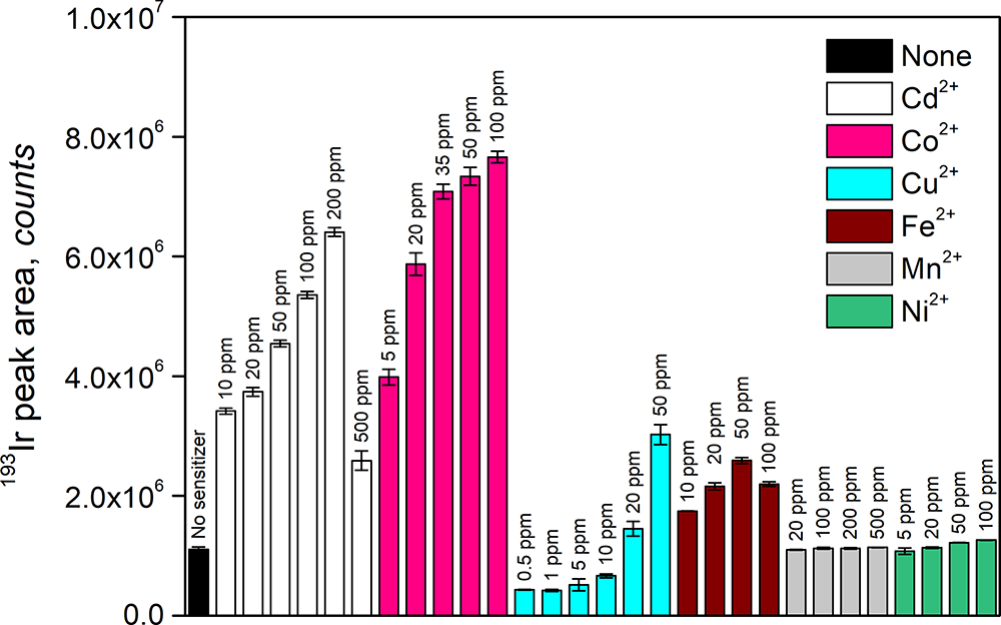
Influence of various metal ion concentrations on PVG response from 100 ng L^–1^ Ir^3+^ in 10 M HCOOH at a sample flow rate of 1.5 mL min^–1^. Uncertainties are expressed as SD (*n* ≥ 3).

The possibility of substantial enhancement yet available in overall PVG efficiency was examined by the addition of various metal ion sensitizers. The metals were added only to the Ir^3+^ standard in 10 M HCOOH and not to the carrier (10 M HCOOH) into which the standard was injected. Metal ions (Cd^2+^, Co^2+^, Cu^2+^, Fe^2+^, and Ni^2+^) were chosen on the basis of their previously reported efficacy on PVG of various analytes.^11,17^ In addition, Mn^2+^, recently reported effective for PVG of Te^4+^, was tested.^36^ The effects of various concentrations of individual metal ions on PVG of Ir at a sample flow rate of 1.5 mL min^–1^ are shown in Figure 2.

The most notable effects were exhibited by Co^2+^ and Cd^2+^ ions, while other ions had significantly less impact. The estimated overall PVG efficiency of ≈30% was achieved using 100 mg L^–1^ Co^2+^, whereas ≈25% was reached using 200 mg L^–1^ Cd^2+^. These respectively constituted approximately 6.9- and 5.8-fold enhancements in comparison to the conditions without sensitizer using only 10 M HCOOH (efficiency typically 4–5%). Higher concentrations of Co^2+^ were not tested so as not to overload the plasma/interface with volatile Co species (see below) and because a plateauing effect seemed to be achieved at concentrations of ≥100 mg L^–1^ Co^2+^.

A quite unique effect was evident for Cu^2+^ in that lower concentrations (0.5–5 mg L^–1^) interfered with PVG of Ir, decreasing the sensitivity by about 2-fold, but at higher concentrations, it curiously enhanced the PVG efficiency. Furthermore, as opposed to other metal ion sensitizers, enhanced production of bubbles was observed when samples containing 20 and 50 mg L^–1^ Cu^2+^ were irradiated. Nevertheless, higher concentrations of added Cu^2+^ were responsible for increased blanks and also memory effects. Copper can be deposited on the conduits of the photoreactor during irradiation^45^ and change irradiation characteristics over time. Note that the impact of added Cu^2+^ observed here is different from that reported by de Oliveira and Borges,^17^ likely because of the significantly different conditions of sample irradiation available with their photoreactor. As for added Fe^2+^, this sensitizer was not particularly effective and maximum overall PVG efficiency of ≈10% was attained at 50 mg L^–1^. Absolutely no or only slight enhancement was observed at 20–500 mg L^–1^ Mn^2+^ and 5–100 mg L^–1^ Ni^2+^.

**Figure 3:**
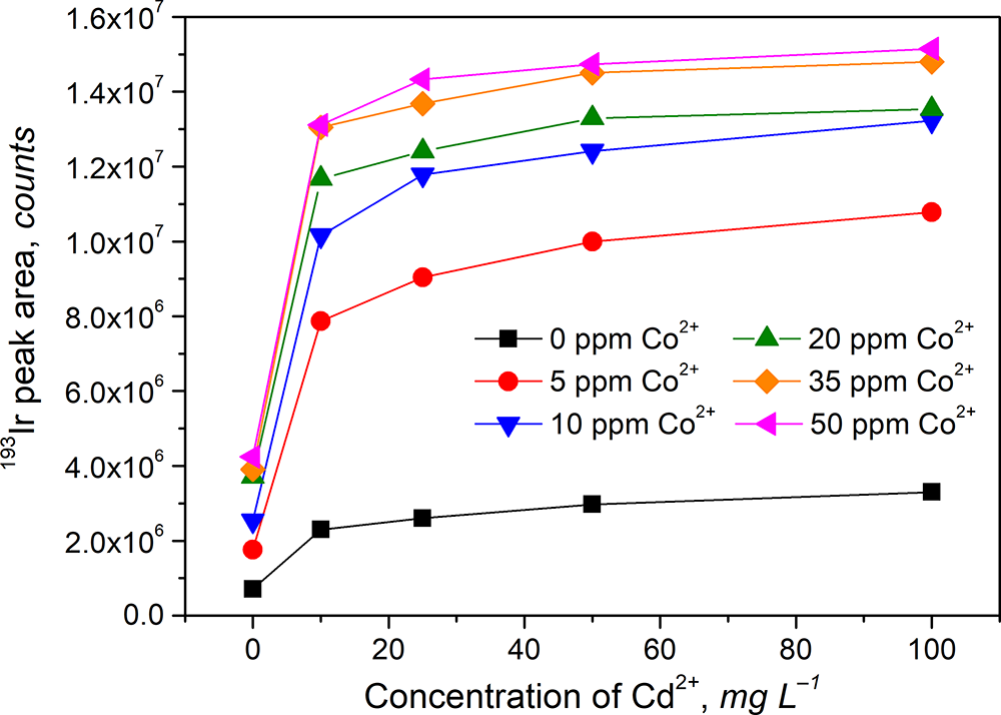
Effect of various combinations of Co^2+^ and Cd^2+^ ions present in 10 M HCOOH on PVG from 50 ng L^–1^ Ir^3+^ at a sample flow rate of 1.5 mL min^–1^. Uncertainties expressed as SD (*n* ≥ 3) are sufficiently small that they cannot be discerned from the data points.

Only Co^2+^ and Cd^2+^ ions appear to be attractive sensitizers for PVG of Ir. Following the recent study of PVG of Ru^3+^, wherein a strong synergistic effect of both ions was identified,^13^ the impact of various combinations of added Co^2+^ and Cd^2+^ sensitizers was further examined, as illustrated in Figure 3.

It is evident that the overall PVG efficiency increases with both Co^2+^ and Cd^2+^ concentrations and when used in combination reach plateaus at 35–50 mg L^–1^ Co^2+^ and 50–100 mg L^–1^ Cd^2+^. The estimate of overall efficiency suggests almost quantitative yield (≥90%) under these PVG conditions. Further experiments also revealed a similar synergistic effect from the co-presence of Fe^2+^ and Cd^2+^ ions wherein a combination of 50 mg L^–1^ Fe^2+^ and 200 mg L^–1^ Cd^2+^ led to an overall PVG efficiency of ≈82%. This combination was not pursued further as the required concentration of Fe^2+^ was too high (see below). Conversely, addition of 50 mg L^–1^ Fe^2+^ to 35 mg L^–1^ Co^2+^ had absolutely no positive influence relative to that from only 35 mg L^–1^ Co^2+^.

A significant concern arises with use of metal sensitizers for PVG in that their volatile species may be co-generated concurrently with the analyte, resulting in high loading of the ICP due to their typically mg L^–1^ concentrations, which is unfavorable for long-term use. With respect to those sensitizers having a significant positive effect, both Co^2+^ and Fe^2+^ result in co-generation of volatile Co(CO)_4_H and Fe(CO)_5_ species, respectively.^46,47^ Negligible and irregular response during PVG was observed for Cd (*m*/*z* 111) if used as the lone sensitizer. The same result was earlier reported for PVG of W sensitized by addition of 500 mg L^–1^ Cd^2+^,^15^ although some contribution from direct PVG action can be expected.^24,33^ The situation slightly improved when Cd^2+^ was used in combination with Co^2+^, but its estimated overall PVG efficiency remained never higher than 0.01%, even when a sample flow rate of 0.75 mL min^–1^ (IT = 58 s) was employed.

### Re-Optimization of the PVG System

A compromise combination of 10 mg L^–1^ Co^2+^ and 25 mg L^–1^ Cd^2+^ was chosen for further experiments to maintain the transfer of Co to the ICP as low as possible. The main PVG conditions, such as HCOOH concentration and IT, were thus re-optimized. The HCOOH concentration was varied in the range of 0.005–16 M HCOOH, revealing a remarkable impact on peak area sensitivity (Figure S5). No dramatic changes in sensitivity were evident over the broad range of 0.1–8 M HCOOH, and only a slight increase toward the maximum at 1 M HCOOH was observed. This dependence is completely different from that shown in Figure S2, wherein a gradual increase in sensitivity occurred with increase in HCOOH concentration up to 18 M for PVG in the absence of sensitizers. Surprisingly, PVG of Ir now remains highly efficient even at very low HCOOH concentrations, wherein the overall efficiency ranges 57–88% for 0.005–0.1 M HCOOH. The Co^2+^ and Cd^2+^ sensitizers are thus extremely beneficial, and their presence significantly alters the course of PVG possibly via an enhancement the yield of highly reducing CO_2_^•–^ during UV irradiation of HCOOH,^11,27,34,35,37^ which likely leads to more efficient reduction of Ir^3+^ to Ir^0^ and subsequent capture of co-generated CO molecules delivered from photolytic homolysis of HCOOH. However, the irritating question arises as to why so little acid remains sufficient to promote such efficient PVG of Ir. A more definitive explanation is not available at this time as the phenomenon requires significantly further investigation.

With respect to such a broad range of suitable HCOOH concentrations for efficient PVG in the presence of sensitizers, the selection of the optimal concentration for routine use and analytical applications presents some complications. Lower concentrations of HCOOH definitely offer some advantages, including lower risk of contamination and less dilution of real water samples with concentrated HCOOH.^39^ Conversely, tolerance toward interferences may be compromised due to a lower concentration of generated CO_2_^•–^ and e_(*aq*)_^–^ as well as less CO for synthesis of Ir carbonyl. Selection of the optimal HCOOH concentration was thus finally based on the tolerance of the system toward the presence of HNO_3_ as it represents the most powerful scavenger of free radicals during PVG of many analytes.^48^ The impact of 0.1–10 mM HNO_3_ at 0.1, 1, 4, and 10 M HCOOH on PVG of Ir is demonstrated in Figure S6. Without doubt, higher tolerance toward HNO_3_ is obtained at higher concentrations of HCOOH. A 4 M HCOOH medium was chosen as a compromise for further experiments, offering reasonable tolerance toward interferences and low sample dilution inevitably accompanying preparation of real samples in HCOOH while also giving rise to a further 7% enhancement in sensitivity over that realized for 10 M HCOOH (cf. Figure 3 and Figure S5).

The dependence of peak area sensitivity on sample flow rate in 4 M HCOOH (Figure S7) became rather featureless, with a plateau in the range of 0.75–1.5 mL min^–1^ (cf. Figure 1 and Figure S4). This indicates that the overall PVG efficiency must be close to 100% (see below) because there is no possibility for further significant improvement using higher ITs.

### Figures of Merit

**Figure 4:**
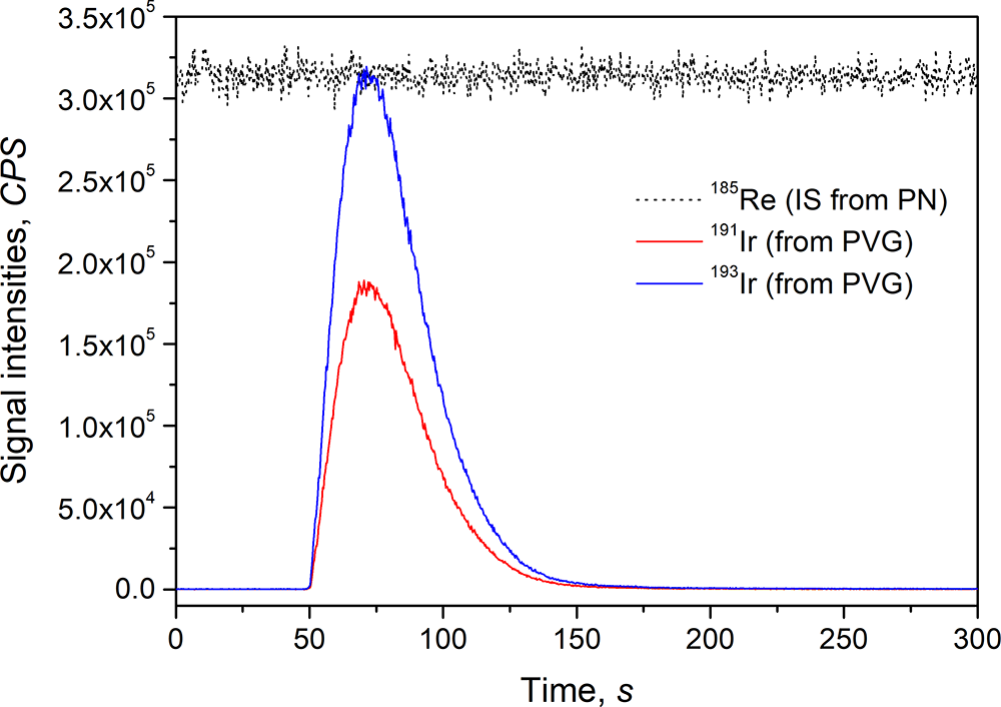
Typical PVG transient signals from 50 ng L^–1^ Ir^3+^ generated from 4 M HCOOH after the addition of 10 mg L^–1^ Co^2+^ and 25 mg L^–1^ Cd^2+^ as sensitizers. Continuous signal of ^185^Re (dotted line) from concurrent nebulization of a solution of 10 μg L^–1^ Re is included for comparison.

The following PVG conditions were selected for routine use: 4 M HCOOH as the reaction medium delivered at 1.5 mL min^–1^ flow rate (IT = 29 s) and the further addition of 10 mg L^–1^ Co^2+^ and 25 mg L^–1^ Cd^2+^ to the standard/sample. A typical FI signal from 50 ng L^–1^ Ir^3+^ is displayed in Figure 4. It is evident from the transients that the FI signals do not suffer from serious tailing, which allows for a satisfactory sampling frequency of 15 samples h^–1^ at concentration levels ≤50 ng L^–1^.

Although the PVG efficiency was estimated daily by comparison of the replicate peak areas measurements of one standard concentration obtained with both FI-PVG and FI-PN processing under the same plasma conditions, the precise value of overall PVG efficiency under the chosen optimal PVG conditions was determined from a comparison of the slopes of calibration functions for FI-PVG (0, 10, 25, and 50 ng L^–1^ Ir^3+^) and FI-PN (0, 50, 250, and 1000 ng L^–1^ Ir^3+^). The sensitivity enhancement factor reached 10.50 ± 0.06, while the nebulization efficiency determined by the dynamic mass flow approach was 8.44 ± 0.04%. These values result in an overall calculated PVG efficiency of 88.6 ± 0.6%. A very similar value of 88.0 ± 2.2% was determined by an indirect approach following an assessment of the Ir remaining in the waste after FI-PVG of a 10 μg L^–1^ solution of Ir^3+^ containing added sensitizers. Although this determination had to be carried out at ≥200-fold higher concentration, it provided supporting evidence that the remaining fraction (11–12%) of Ir is not firmly deposited in the conduits of the generator.

An increase to 91–95% can be easily obtained at lower HCOOH concentrations (1–2 M; Figure S5) or with the use of higher concentrations of metal sensitizers, especially Co^2+^ (Figure 3). An overall PVG efficiency of 97.5 ± 0.7% can also be achieved if the sensitizers (10 mg L^–1^ Co^2+^ and 25 mg L^–1^ Cd^2+^) are added not only to the Ir^3+^ standard in 4 M HCOOH but also to the reaction medium serving as the carrier. In the case when an Ir^3+^ standard containing sensitizers is injected into a carrier stream comprising only 4 M HCOOH, dispersion of the analyte as well as metal sensitizers in the photoreactor conduit cannot be avoided and the concentrations of metal sensitizers at the leading and falling edges of the analyte zone become lower during their transport through the photoreactor. Nevertheless, the potential slight increase in overall PVG efficiency is not worth incurring problems associated with overloading the plasma with high metal concentrations of continuously co-generated Co.

The PVG methodology is characterized by excellent precision (RSD); a repeatability of 1.0% (*n* = 15) in the peak area was obtained for a quality control check standard of 50 ng L^–1^ Ir^3+^ used throughout the course of one measurement day. All calibration functions for both ^191^Ir and ^193^Ir isotopes using 0, 1, 2, 4, 10, 25, and 50 ng L^–1^ Ir^3+^ standards measured using either a no gas mode or He mode in the reaction/collision cell were linear (*R*^2^ > 0.9999). Sensitivities in He mode were approximately 35% lower than those in the no gas mode due to ion scattering. Using no gas mode, outstanding LODs (3σ, *n* = 11) of 3 pg L^–1^ (1.5 fg absolute) were reached for both ^191^Ir and ^193^Ir, whereas using He mode provided LODs (*n* = 11) of 6 pg L^–1^ (3 fg absolute). A typical small but persistent peak-shaped signal comprising single to a few tens of counts for 0.1 s dwell time characterized blank measurements. The LODs are thus driven by the variance of the peak area of this blank signal, the concentration of which corresponded to 0.015–0.03 ng L^–1^ provided that the PVG system was clean and not contaminated by prior measurement of a sample containing >10 ng L^–1^ Ir. The source of this blank was, in part, derived from the added Co^2+^ sensitizer solution. Sub-boiling distillation of HCOOH or use of ultrapure water from a commercial supplier provided no improvement in blank values.

The influence of the oxidation state of Ir (Ir^3+^ vs Ir^4+^) on PVG efficiency was also examined using standards prepared from solid (NH_4_)_2_[IrCl_6_], i.e., Ir^4+^, and (NH_4_)_3_[IrCl_6_], i.e., Ir^3+^. Compared to the sensitivity obtained using a commercial analytical standard (as IrCl_3_), an identical response (100 ± 2%) was verified with Ir solutions prepared using both solid salts. The redox stability of these Ir species in prepared reaction media (4 M HCOOH with or without sensitizers) was examined by UV–vis spectrometry based on use of 5 mg L^–1^ Ir solutions. No decline in intensity of the absorption maxima at 305, 418, 435, and 488 nm, indicating the presence of [IrCl_6_]^2–^,^49^ was identified over the course of 1 week and vice versa, and no appearance of such bands was found for aging a similar Ir^3+^ standard. Hence, the redox stability of the Ir species in the prepared reaction media containing sensitizers seems not to be an issue and the sequential reduction of Ir^4+^ to Ir^3+^ to Ir^0^ can be confirmed as due to the action of PVG.

For comparison, LODs (*n* = 13) for conventional PN-ICP-MS/MS employing an autosampler and continuous flow steady-state measurements were evaluated for ^191^Ir and ^193^Ir. Using standard no gas mode, 0.07 and 0.06 ng L^–1^, respectively, were obtained, whereas in He mode, these values were 0.09 and 0.06 ng L^–1^, respectively. It is evident that FI-PVG-ICPMS surpasses conventional PN-ICPMS/MS in terms of LODs by more than one order of magnitude, which arises as a result of the more than 10-fold increase in the efficiency of analyte introduction into the plasma.

The substantial performance benefits arising from PVG of Ir are a direct consequence of its introduction as a gas phase species.^50^ It is assumed that the volatile species synthesized herein may be Ir(CO)_4_H, Ir_2_(CO)_8_ or Ir_4_(CO)_12_ based on the use of HCOOH giving rise to only hydrided/carbonylated adducts. In relatively older studies, formation of Ir(CO)_4_H was reported when water containing IrCl_3_ was used for the preparation of other iridium carbonyls and detected as a very volatile compound,^51^ including the direct evidence by Whyman,^52^ who used IR spectroscopy to investigate reactions of Ir_4_(CO)_12_ under various pressures of CO and H_2_ and found no evidence for formation of Ir_2_(CO)_8_.^53^ To the best of our knowledge, successful GC–MS detection of any iridium carbonyl has not yet been achieved (including our most recent attempts),^54^ and there is also no MS spectrum available in the NIST database.^55^ Hence, identification of the product of PVG may be challenging despite its relatively good stability based on experience generated in this study.

### Interferences

**Figure 5:**
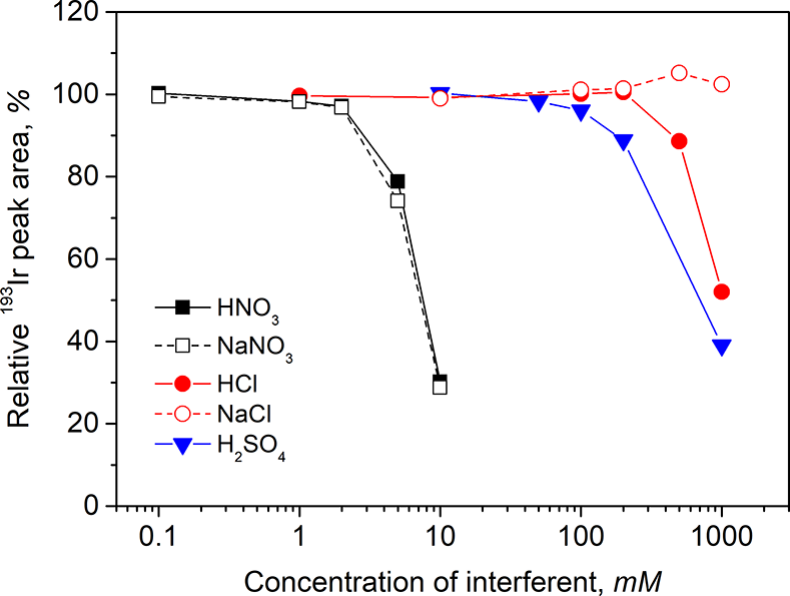
Relative effects of added inorganic acids and salts on PVG from 50 ng L^–1^ Ir^3+^ prepared in 4 M HCOOH containing 10 mg L^–1^ Co^2+^ and 25 mg L^–1^ Cd^2+^ as sensitizers. Combined uncertainty associated with individual data points is lower than 2% in all cases.

Interference effects from HNO_3_, HCl, and H_2_SO_4_ arising under compromised optimal PVG conditions were examined. Consideration of their possible application with the proposed methodology arises from their use during digestion and/or stabilization of samples. Figure 5 shows that a serious suppressive effect occurs for HNO_3_, wherein concentrations of 5 and 10 mM cause 26 and 71% decreases in sensitivity, respectively. (Note: The same dependence is actually presented in Figure S6, where tolerance toward HNO_3_ was compared for various concentrations of HCOOH). This interference seems to be driven by the NO_3_^–^ anion because the impact of added NaNO_3_ salt was completely identical. The PVG system was, by almost two orders of magnitude, more tolerant to the presence of H_2_SO_4_ in that a significant decrease in sensitivity (by 11%) occurred at 200 mM. Tolerance toward Cl^–^ anion was excellent, which can be demonstrated by a significant decrease (>10%) in response only at concentrations higher than 500 mM (for HCl) and by absolutely no negative interference from NaCl up to 1 M. The lower tolerance toward HCl compared to NaCl is likely caused by a concurrent change in sample pH. To the best of our knowledge, such excellent tolerance toward HCl and NaCl has not been reported for PVG of any other transition metal and encourages a direct determination of dissolved Ir in complex matrices such as seawater (≈0.54 M Cl^–^) without any required dilution.

In addition to inorganic acids, the effect of potential co-existing transition metals that can be present in typical prepared samples of interest containing Ir (i.e., Mn^2+^, Fe^3+^, Cu^2+^, Zn^2+^, Mo^6+^, Rh^3+^, Pd^2+^, Pt^4+^, and Au^3+^) and metalloids (As^3+^ and Se^4+^) was examined in the range of 0.01–10 mg L^–1^ (see Table S3 in the Supporting Information). No negative interference was found for Mn^2+^, Fe^3+^, Zn^2+^, and Mo^2+^ present in prepared samples at levels up to 10 mg L^–1^. With the exception of Pd^2+^ inducing a 13% suppression in response at only 0.1 mg L^–1^, a significant decrease in sensitivity (>10%) was identified for other elements at 1 mg L^–1^ levels, namely, by 20% for Cu^2+^, 70% for As^3+^, 31% for Se^4+^, 36% for Pt^4+^, and 42% for Au^3+^. It was remarkable that the negative interference from Cu^2+^ ions (around 50%) observed at 0.5–5 mg L^–1^ during the assessment of potential sensitizers (Figure 2) was alleviated under conditions arising with the recommended sensitizers; recovery in the presence of 10 mg L^–1^ Cu^2+^ actually increased to 91%. This behavior is in line with the already described effect of higher concentrations of Cu^2+^ on PVG without added Co^2+^ and Cd^2+^ as sensitizers.

### Application to Real Samples

Verification of the accuracy of the developed methodology was challenging due to an absolute lack of certified reference materials with specified Ir content, likely because Ir concentrations in common environmental samples are below detection capabilities of the majority of analytical methods. It is possible that Ir may have become a new component of auto catalytic converters, such as iridium-containing units for direct injection engines manufactured by Mitsubishi of Japan.^4^ NIST SRM 2556 (Used Auto Catalyst) was thus chosen to validate the accuracy of the developed methodology. SRM 2556 is certified only for Pb and other PGEs, i.e., Pt (697.4 mg kg^–1^), Pd (326 mg kg^–1^), and Rh (51.2 mg kg^–1^), but Vobecký et al.^56^ earlier reported an estimate of 19 μg kg^–1^ Ir by instrumental activation analysis when utilizing a coincidence spectrometer. As Ir may be present in SRM 2556 as a metallic impurity that is resistant to attack by all acids, including aqua regia, sample preparation excludes the use of conventional wet acid digestion techniques for solubilization.^57^ Peroxide fusion at 620 °C was thus applied for sample preparation. Fused Na_2_O_2_ oxidizes Ir to IrO_2_ that is subsequently dissolved in dilute HCl and converted to [IrCl_6_]^4–^, resulting in an overall 125-fold dilution of the sample (see the Experimental Section). As discussed earlier, the presence of Ir^4+^ species is irrelevant for PVG, and Ir^3+^ standards can be confidently employed for calibration and determination of total Ir by FI-PVG-ICPMS. An aliquot of the digest was diluted a further 100-fold with 4 M HCOOH containing 10 mg L^–1^ Co^2+^ and 25 mg L^–1^ Cd^2+^ as sensitizers and subjected to FI-PVG-ICPMS using He mode detection. The Ir content was quantified by external calibration and corrected for total digestion blank yielding 20.1 ± 0.1 μg kg^–1^, whereas 20.9 ± 0.2 μg kg^–1^ was obtained by use of the standard additions technique (comprising 1 and 2 ng L^–1^ spiked concentrations), confirming good recovery of 96 ± 1%. It is noteworthy that direct analysis of the digested diluted matrix presented by SRM 2556 can be undertaken using calibration against external standards because the concentrations of all potential interfering ions are at levels at which they exert no significant impact (Table S3). (Note: Final concentrations of 56 μg L^–1^ for Pt, 26 μg L^–1^ for Pd, and 4 μg L^–1^ for Rh can be expected taking into account a total dilution factor of 12,500).

For comparison, a second aliquot of the sample digest was diluted 20-fold with 2% (m/v) HNO_3_, yielding a solution containing ≈0.3% dissolved NaCl, which was subjected to determination by conventional PN-ICP-MS/MS using both no gas and He modes of the reaction/collision cell and matrix matched calibration. Values of 21.4 ± 1.0 μg kg^–1^ and 21.4 ± 1.2 μg kg^–1^ were obtained using ^191^Ir for evaluation in no gas and He mode, respectively, whereas significantly higher values of 29.2 ± 0.8 and 26.9 ± 2.0 μg kg^–1^ resulted when using ^193^Ir for evaluation. The reason for this discrepancy is likely due to relatively high Pt content in SRM 2556 causing tailing (abundance sensitivity) interferences from adjacent ^194^Pt (natural abundance 32.97%) and ^192^Pt (0.782%), especially affecting the ^193^Ir isotope despite the “high” resolution setting of ICPMS/MS used (see Table S2). It should be noted that this type of spectral interference does not occur with FI-PVG because the PVG efficiency for Pt at 1 μg L^–1^ under conditions optimal for Ir was found to be negligible (≈0.23%). Interestingly, the PVG efficiencies of the other two PGEs present at high concentrations and certified in SRM 2556 were also tested and found to be very low, reaching ≈0.07% for Pd and ≈0.16% for Rh. With the exception of the result obtained with conventional PN-ICP-MS/MS using ^193^Ir for evaluation, the results for Ir content in SRM 2556 agree very well with the earlier value reported by Vobecký et al.^56^

The practical feasibility of determination of dissolved Ir (as Ir^3+^ and Ir^4+^) at extremely low concentrations was also examined by a direct analysis of five water samples of different matrix complexity. No dilution of the water samples was required except for the addition of concentrated HCOOH and solutions of sensitizers (final dilution factor of only 1.19). The water samples were analyzed by FI-PVG-ICPMS in He mode. Recoveries were calculated from the ratio of slopes of the standard additions (0, 1 and 2 ng L^–1^ Ir spikes added) versus external calibration functions (0, 1 and 2 ng L^–1^ standards). Excellent recovery in the range of 99–102% was achieved for all five samples (Table 1), including a seawater that contains ≈0.54 M Cl^–^. This is in line with the data presented in Figure 5, wherein a tolerance to NaCl is demonstrated even up to 1 M. In parallel, the determination of Ir was attempted by conventional PN sample introduction ICPMS/MS. Results confirmed undetectable levels of dissolved Ir in spring, river, and lake water (below LODs). Seawater samples could not be examined via PN introduction due to the necessity of additional dilution, resulting in further degradation of LODs.

**Table 1:** FI-PVG-ICPMS Determination of Dissolved Ir in Water Samples

samples	found (ng L^–1^)	recovery^a^
spring water	<0.007^b^	99 ± 2
river water	<0.007^b^	100 ± 1
lake water	<0.007^b^	99 ± 1
seawater I	0.007^b^ < *x* < 0.024^c^	102 ± 1
seawater II	0.007^b^ < *x* < 0.024^c^	99 ± 2

a Spike recovery = slope of standard additions (no addition, 1 and 2 ng L^–1^ spiked to a sample prepared in the reaction medium containing sensitizers)/slope of external calibration (0, 1, and 2 ng L^–1^) × 100 (%).

b LOD = 0.007 ng L^–1^ (0.006 ng L^–1^ corrected for dilution factor of 1.19 introduced by preparation of water sample in the reaction medium).

c LOQ = 0.024 ng L^–1^ (0.020 ng L^–1^ corrected for dilution factor of 1.19 introduced by preparation of water sample in the reaction medium).

## Conclusions

High PVG efficiency of Ir was achieved under various conditions. If conducted in the absence of metal ion sensitizers, excessive IT appears necessary, which is not feasible using a flow setup; a “stop-flow” mode of operation could be more suitable for this purpose. A substantial enhancement in PVG can be obtained by increasing pH of the reaction medium, although this still requires a lengthy IT or by addition of Co^2+^ and Cd^2+^ ions as sensitizers which synergistically enhance the process. The latter approach was found more practical for analytical applications and also led to an overall PVG efficiency close to 100%.

Excellent repeatability and LODs in the range of 3–6 pg L^–1^ were achieved. These LODs are without doubt the best reported for any element using any VG technique without resorting to prior preconcentration. In addition, excellent tolerance toward chloride anion predestines this method for direct analyses of matrices that would otherwise require significant dilution preceding determination by conventional PN-ICPMS or that of samples following peroxide fusion necessitated for dissolution of metallic Ir.

The variety of real samples selected for analysis in this study was limited in scope due to the non-availability of matrix reference materials certified for Ir content. Consequently, corresponding interference studies have likewise been constrained to reflect the composition of only the real samples examined. Appropriately, it is incumbent on the users of this PVG methodology to first verify the recovery of spikes so as to determine the severity of potential interference encountered with uncharacterized matrices before resorting to use of external calibration for quantitation. Both the method of additions as well as isotope dilution approaches may be confidently used as spectral interferences should be absent.

## Supporting Information Available

Details on instrumentation; scheme of PVG arrangement coupled to ICPMS; ICPMS parameters for FI-PVG and for conventional PN used for real sample analysis; experiments dealing with release and transport of volatile species; effect of HCOOH concentration without additives; effect of pH and sample flow at pH = 3.4 of the reaction medium; influence of HCOOH concentration and sample flow rate using Co^2+^ and Cd^2+^ as sensitizers; relative effects of added HNO_3_ in various HCOOH media containing Co^2+^ and Cd^2+^ as sensitizers; influence of various co-existing ions (PDF)
