# Supplementary material for: Highly Efficient Photochemical Vapor Generation for Sensitive Determination of Iridium by Inductively Coupled Plasma Mass Spectrometry
Source: Anal Chem. 2023 Feb 10;95(7):3694–702. doi: 10.1021/acs.analchem.2c04660 (PMC10016747; doi:10.1021/acs.analchem.2c04660)
Supplement: Supplementary file 1 — ac2c04660_si_001.pdf [file ac2c04660_si_001.pdf]

Supporting Information

Highly Efficient Photochemical Vapor Generation for Sensitive Determination of Iridium by Inductively Coupled Plasma Mass Spectrometry

Stanislav Musil,[a,*] Eva Jeníková [a,b] Jaromír Vyhnanovský,[a,b] and Ralph E. Sturgeon[c]

[a] Institute of Analytical Chemistry of the Czech Academy of Sciences, Veveří 97, 602 00 Brno, Czech Republic

[b] Charles University, Faculty of Science, Department of Analytical Chemistry, Hlavova 8, 128 43 Prague, Czech Republic

[c] National Research Council of Canada, 1200 Montreal Road, Ottawa, Ontario K1A 0R6, Canada

* Corresponding author; E-mail: stanomusil@biomed.cas.cz (S. Musil)

TABLE OF CONTENTS:

Details on instrumentation.. S-3

Figure S1. PVG arrangement for FI coupling to ICPMS with simultaneous liquid nebulization... S-4

Table S1. Typical ICPMS/MS parameters for coupling with PVG...................................... S-5

Table S2. ICPMS/MS parameters for conventional PN sample introduction........................ S-5

Experiments dealing with release and transport of volatile species...................................... S-6

Figure S2. Effect of HCOOH concentration without additives.. S-7

Figure S3. Effect of pH of reaction medium... S-7

Figure S4. Effect of sample flow rate at pH = 3.4 of reaction medium................................ S-8

Figure S5. Influence of HCOOH concentration using Co^{2+} and Cd^{2+} as sensitizers............. S-8

Figure S6. Relative effects of added HNO_3 in various HCOOH media containing Co^{2+} and Cd^{2+} as sensitizers.. S-9

Figure S7. Effect of sample flow rate using Co^{2+} and Cd^{2+} as sensitizers in 4 M HCOOH... S-9

Table S3. Influence of various co-existing ions on Ir response... S-10

References.. S-10

EXPERIMENTAL SECTION

Instrumentation. Sample solutions were introduced in a flow-injection (FI) mode into a stream of the reaction medium with the aid of an injection valve (0.5 mL sample volume). Delivery at an arbitrary flow rate to the photoreactor was undertaken using a peristaltic pump (Reglo Digital, Ismatec) which was also used to evacuate waste from the gas-liquid separator (GLS). All connecting tubing was made of PTFE (i.d. 1 mm) with the exception of the Tygon pump tubing. The high-efficiency flow-through photoreactor was a 19 W low-pressure mercury discharge lamp (Jitian Instruments Co., Beijing, China) internally fitted with three efficiently irradiated lengths of synthetic quartz tubing (total volume 0.72 mL) as well as two short quartz segments on either end of the photoreactor (≈0.25 mL) which serve as exterior inlet and outlet sample connection ports; these segments are not efficiently irradiated. The effluent was mixed with a flow of Ar carrier and directed to the GLS (15 mL internal volume), as described elsewhere.[1,2] The outlet of the GLS was connected via PTFE tubing (2 mm i.d. x 40 cm long) to an Agilent 8900 ICPMS/MS via an ultra-high matrix introduction (UHMI) port located downstream of the Scott double-pass spray chamber of the ICPMS. This port is originally intended for on-line dilution of aerosols of samples with high salt content. Carrier liquid (2% (m/v) HNO_3), mixed with an internal standard (IS) solution of 10 µg L^{-1} Re in 2% (m/v) HNO_3, was concurrently introduced into the spray chamber via a MicroMist nebulizer (Burgener Research Inc., Mississauga, Canada). The liquid carrier channel was equipped with a manual injection valve (0.5 mL sample loop volume) and this arrangement was exclusively utilized for estimation/determination of overall PVG efficiency (see Section "Procedure and Conventions"). A schematic of the PVG system coupled to ICPMS is depicted in Figure S1.

No special cleaning of the photoreactor was necessary between sequential measurements and the chemifold was typically only flushed with DIW at the end of the measurement day. From time to time, the quartz photoreactor was manually filled with concentrated HNO_3 via a syringe and the UV lamp was powered on to initiate decomposition of HNO_3 and to facilitate dissolution and removal of any deposited (metal) impurities.

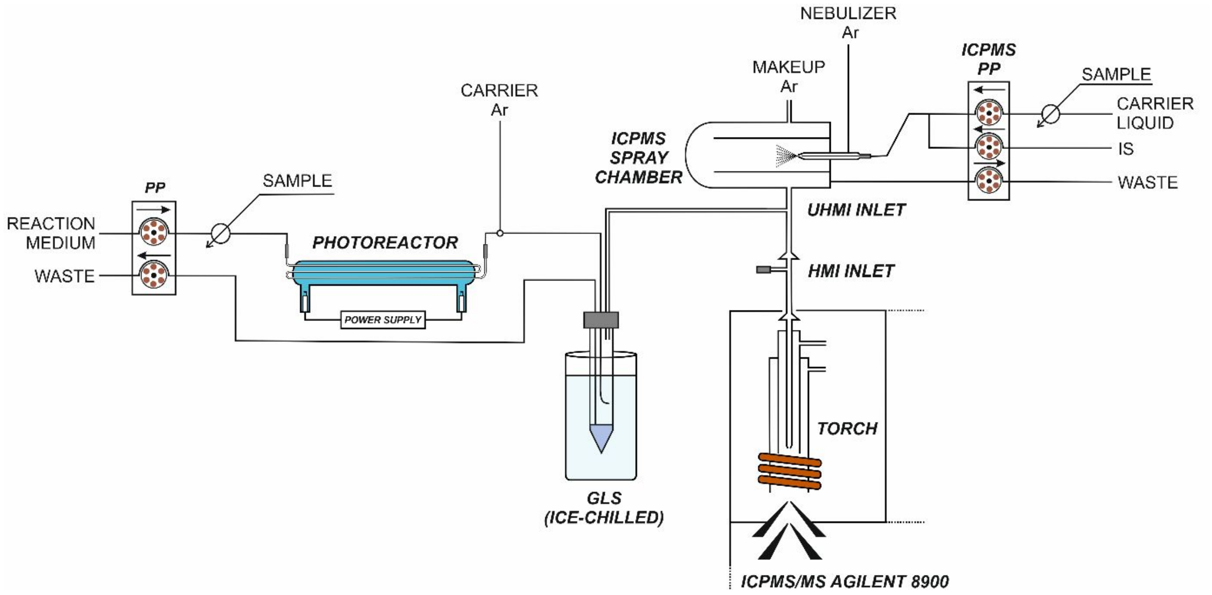

Figure S1. PVG arrangement for FI coupling to ICPMS with simultaneous liquid nebulization. GLS – gas-liquid separator, UHMI – ultra-high matrix introduction port.

Detection of generated volatile Ir species was achieved using an Agilent 8900 triple quadrupole ICPMS employing "wet plasma" conditions created by simultaneous pneumatic nebulization (PN) of a carrier liquid (2% (m/v) HNO_3) mixed on-line with the IS solution.[1-4] More robust conditions are created in the ICP and this setup permits the monitoring/correction for any sensitivity drift due to changes in the plasma or interface transmission efficiency by means of the response changes noted for the nebulized IS.

Optimal plasma settings of the Agilent 8900 ICPMS/MS for coupling with PVG are summarized in Table S1. Isotopes of ^{185}Re (IS, dwell time 0.05 s), ^{191}Ir (0.1 s) and ^{193}Ir (0.1 s) were always monitored and accompanied by ^{55}Mn (0.001 s), ^{56}Fe (0.001 s), ^{59}Co (0.001 s), ^{63}Cu (0.001 s), ^{103}Rh (0.05), ^{105}Pd (0.05), ^{111}Cd (0.001 s), ^{192}Pt (0.05 s) or ^{194}Pt (0.05 s) according to the actual demands during the optimization or real sample analysis. The transient FI signals were exported to, and integrated with, MS Excel and corrected for any sensitivity drift relative to changes in the co-introduced ^{185}Re IS signal intensity.

Table S1. Typical ICPMS/MS (Agilent 8900) parameters for coupling with PVG

RF power	1,550 W
RF matching	1.4 V
Sampling depth	8.0 mm
Nebulizer Ar	960 mL min^{-1}
Makeup Ar	0 mL min^{-1}
Carrier Ar for PVG	200 mL min^{-1}
ICPMS peristaltic pump flow	0.1 rps (0.33 mL min^{-1} carrier liquid, 0.06 mL min^{-1} IS)
Spray chamber temperature	2 °C
Reaction/collision cell mode	No gas or He (4.1 mL min^{-1})
Acquisition mode	Time-resolved analysis
Scan type	Single quad
Measured isotopes (dwell time, s)[a]	^{191}Ir (0.1), ^{193}Ir (0.1), ^{185}Re (IS, 0.05)

[a] other isotopes, in addition to the default ones, monitored during the optimization or real sample analysis included: ^{55}Mn (0.001 s), ^{56}Fe (0.001 s), ^{59}Co (0.001 s), ^{63}Cu (0.001 s), ^{103}Rh (0.05), ^{105}Pd (0.05), ^{111}Cd (0.001 s), ^{192}Pt (0.05 s) or ^{194}Pt (0.05 s)

Conventional PN-ICPMS/MS equipped with an Agilent SPS 4 autosampler was employed for comparative determination of Ir in SRM NIST 2556 (Used Auto Catalyst) after peroxide fusion as well as for real water samples and to evaluate LODs for comparison with those by PVG. Five replicate steady-state measurements were acquired using both a standard no gas mode and He mode (4.1 mL min^{-1}) of the collision cell. The gas flow, MS settings and measured isotopes are summarized in Table S2.

Table S2. ICPMS/MS parameters for conventional PN sample introduction

RF power	1,550 W
RF matching	1.2 V
Sampling depth	8.0 mm
Nebulizer Ar	600 mL min^{-1}
Dilution Ar (UHMI)	550 mL min^{-1}
ICPMS peristaltic pump flow	0.1 rps (0.33 mL min^{-1} carrier liquid, 0.06 mL min^{-1} IS)
Spray chamber temperature	2 °C
Reaction/collision cell mode	No gas and He (4.1 mL min^{-1})
Acquisition mode	Spectrum (5 replicates)
Scan type	MS/MS (narrow peak)
Measured isotopes (dwell time, s)	^{191}Ir (0.3), ^{193}Ir (0.3), ^{101}Ru (IS, 0.1), ^{185}Re (IS, 0.1)

RESULTS AND DISCUSSION

Release and Transport of Volatile Species. The effect of argon (chemifold) carrier flow on the release and transport of volatile species to the ICPMS was examined using a reaction medium of 10 M HCOOH and sample flow rate of 1.5 mL min^{-1}. The gas stream leaving the GLS was mixed with an additional flow of argon (not shown in Figure S1) before it was introduced to the ICPMS. Care was taken to keep the total gas flow to the ICP the same, so as not to influence conditions in the plasma or sampling depth. Although a slightly lower peak area sensitivity (by 9%) was obtained at 50 mL min^{-1} supplied to the GLS, no significant further effect of carrier Ar for PVG was observed in the range 100−600 mL min^{-1}, suggesting an efficient release and stability of the gaseous product. A flow rate of 200 mL min^{-1} was chosen as optimal in order to minimize any potential load of HCOOH vapor on the ICP.

The efficiency of release of volatile species from the liquid reaction medium was also investigated. The GLS was operated in such a way that no liquid comprising the sample was maintained in the GLS (the PTFE tube for waste removal was moved to the bottom of the GLS) and the peak area sensitivities were compared to those obtained with 1.5 mL of the reaction medium maintained inside the GLS. No significant changes in peak area sensitivity using 10 M HCOOH as the reaction medium were identified between either setup for sample flow rates of 1, 1.5 and 2.5 mL min^{-1}. This result suggests that the majority of the volatile Ir species is released to the gas phase prior to reaching the GLS, most probably after mixing with carrier Ar in the short transfer line to the GLS (see Figure S1) or the gas-liquid partitioning of the volatile species is intrinsically rapid. Chilling of the GLS in an ice-water bath also had no impact on peak area sensitivity but was used throughout as it significantly limited carryover of small droplets of the reaction medium formed in the GLS to the transport tube connected to the ICP and thus improved stability of measured signals.

PVG without Additives

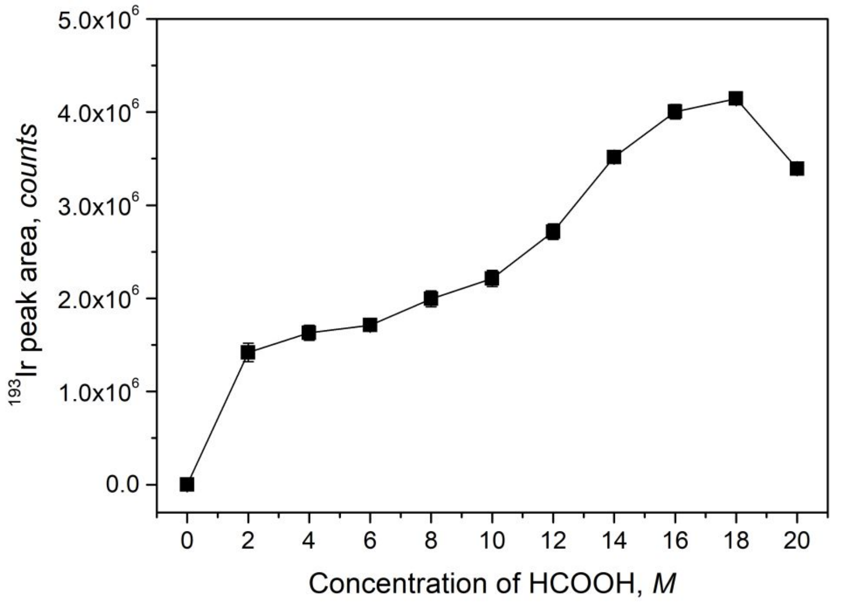

Figure S2. Influence of HCOOH concentration on peak area response from 200 ng L^{-1} Ir^{3+} at a sample flow rate of 1.5 mL min^{-1}. Uncertainties expressed as SD ($n \geq 3$) are sufficiently small that they cannot be discerned from the data points in some cases.

Effect of pH

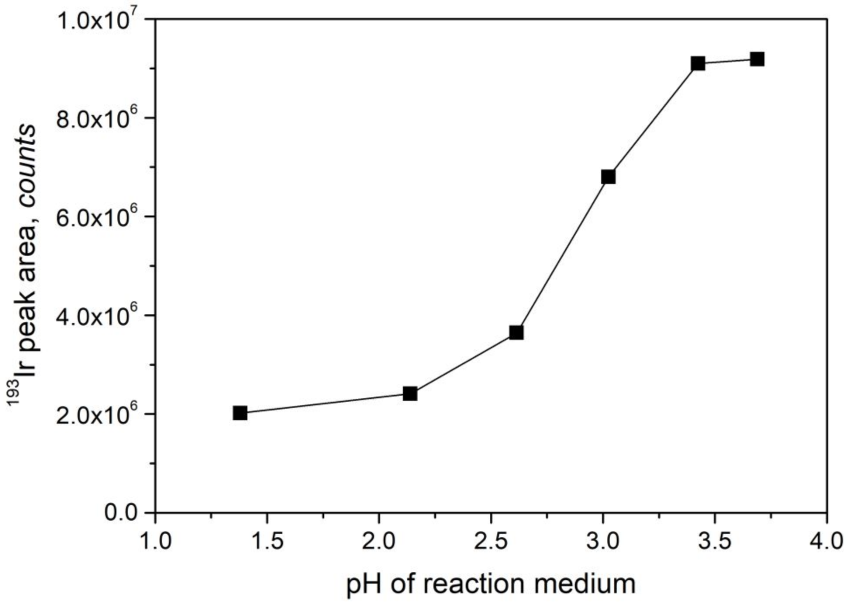

Figure S3. Effect of pH of the reaction medium on peak area response from 200 ng L^{-1} Ir^{3+} at a sample flow rate of 1.5 mL min^{-1}. pH was adjusted by varying the volume of liquid $NH_3{\cdot}H_2O$ added to 10 M HCOOH. Uncertainties expressed as SD ($n \geq 3$) are sufficiently small that they cannot be discerned from the data points.

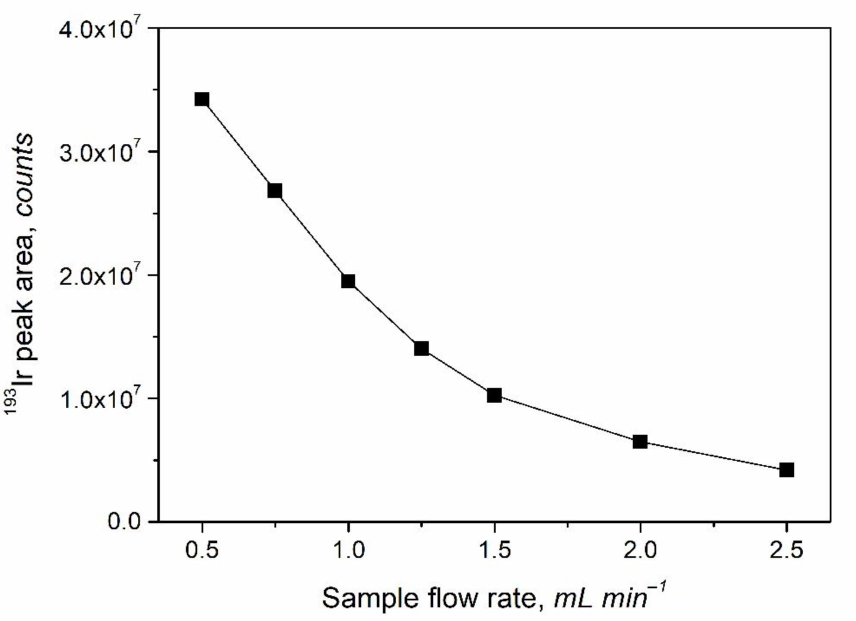

Figure S4. Effect of sample flow rate using 4 M $HCOONH_4$ in 6 M HCOOH as the reaction medium (pH = 3.4) on peak area response from 200 ng L^{-1} Ir^{3+}. Uncertainties expressed as SD (n ≥ 3) are sufficiently small that they cannot be discerned from the data points.

Re-optimization of the PVG System

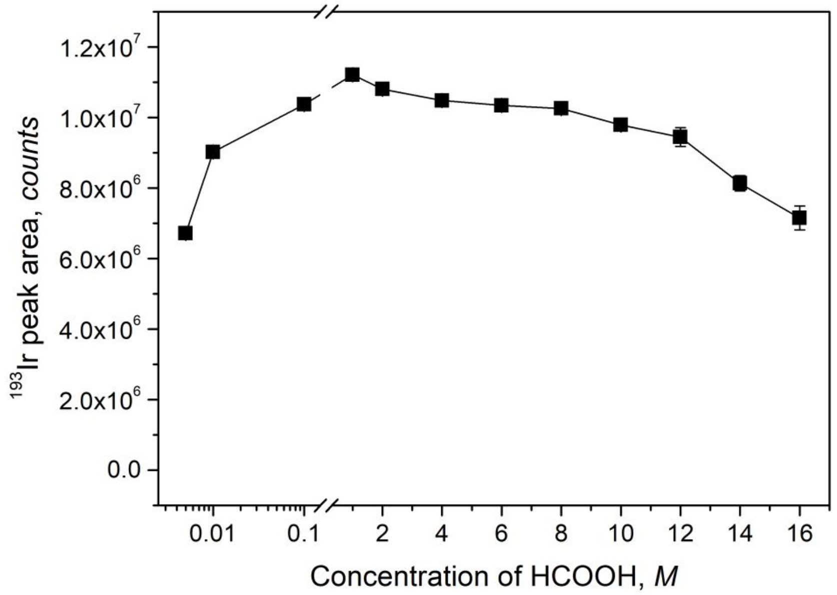

Figure S5. Influence of HCOOH concentration on peak area response from 50 ng L^{-1} Ir^{3+} using 10 mg L^{-1} Co^{2+} and 25 mg L^{-1} Cd^{2+} as sensitizers at a sample flow rate of 1.5 mL min^{-1}. The range 0.0025 M to 0.15 M given in logarithmic scale. Uncertainties expressed as SD (n ≥ 3) are sufficiently small that they cannot be discerned from the data points in some cases.

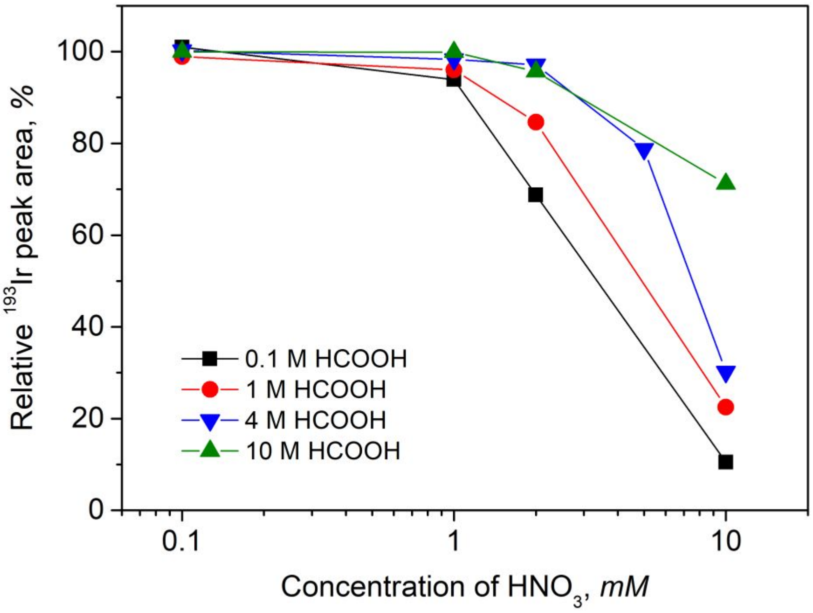

Figure S6. Relative effects of added HNO_3 on PVG from 50 ng L^{-1} Ir^{3+} in various HCOOH media containing 10 mg L^{-1} Co^{2+} and 25 mg L^{-1} Cd^{2+} as sensitizers. Combined uncertainty associated with individual data points is lower than 2% in all cases.

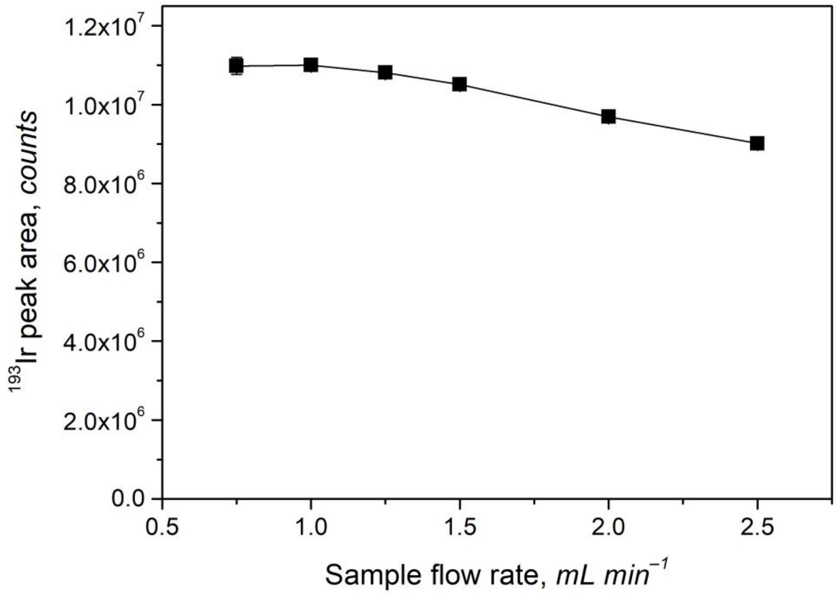

Figure S7. Effect of sample flow rate using 10 mg L^{-1} Co^{2+} and 25 mg L^{-1} Cd^{2+} as sensitizers in 4 M HCOOH on peak area response from 50 ng L^{-1} Ir^{3+}. Uncertainties expressed as SD (n ≥ 3) are sufficiently small that they cannot be discerned from the data points in some cases.

Interferences

Table S3. Influence of various co-existing ions (expressed as % recovery)[a] on Ir response (50 ng L^{-1}) examined by FI-PVG-ICPMS

Interferent	Concentration (mg L^{-1})			
	0.01	0.1	1	10
Mn^{2+}	98	99	99	99
Fe^{3+}	98	99	98	101
Cu^{2+}	99	97	80	91
Zn^{2+}	99	100	100	99
As^{3+}	98	90	30	1
Se^{4+}	100	92	69	25
Mo^{6+}	99	101	99	99
Rh^{3+}	99	96	70	14
Pd^{2+}	101	87	19	3
Pt^{4+}	100	93	64	34
Au^{3+}	99	100	58	13

[a] associated relative combined uncertainty (combined uncertainty/recovery) is lower than 2% for all calculated recovery values

REFERENCES

(1) Vyhnanovský, J.; Sturgeon, R. E.; Musil, S. Cadmium Assisted Photochemical Vapor Generation of Tungsten for ICPMS detection. *Anal. Chem.* **2019**, *91* (20), 13306–13312. DOI: 10.1021/acs.analchem.9b04241

(2) Vyhnanovský, J.; Yildiz, D.; Štádlerová, B.; Musil, S. Efficient photochemical vapor generation of bismuth using a coiled Teflon reactor: Effect of metal sensitizers and analytical performance with flame-in-gas-shield atomizer and atomic fluorescence spectrometry. *Microchem. J.* **2021**, *164*, 105997. DOI: 10.1016/j.microc.2021.105997

(3) Musil, S.; Vyhnanovský, J.; Sturgeon, R. E. Ultrasensitive Detection of Ruthenium by Coupling Cobalt and Cadmium Ion-Assisted Photochemical Vapor Generation to Inductively Coupled Plasma Mass Spectrometry. *Anal. Chem.* **2021**, *93* (49), 16543–16551. DOI: 10.1021/acs.analchem.1c03739

(4) Šoukal, J.; Sturgeon, R. E.; Musil, S. Efficient Photochemical Vapor Generation of Molybdenum for ICPMS Detection. *Anal. Chem.* **2018**, *90* (19), 11688–11695. DOI: 10.1021/acs.analchem.8b03354
